# Supplementary material for: Exploring the recurrent states of football teams’ tactical organization on the pitch during Brazilian official matches
Source: PLoS One. 2024 Aug 12;19(8):e0308320. doi: 10.1371/journal.pone.0308320 (PMC11318918; doi:10.1371/journal.pone.0308320)
Supplement: S1 Appendix — (ZIP) [file pone.0308320.s001.zip › S1_Appendix.pdf]

S1 Appendix Recurrence Quantitative Analysis Results

Appendix Table A.3. Recurrence Quantitative Analysis measures considering the **whole match**.

Team	RR	DET	Lmax	L	ENTR	LAM	TT
1	59.4	46.0	21.0	3.9	1.9	62.0	4.6
2	58.2	45.6	19.0	4.0	1.9	62.8	4.6
3	52.6	46.3	18.0	4.0	2.0	63.8	4.7
4	51.9	45.2	23.0	4.0	2.0	63.1	4.7
5	60.3	46.1	30.0	4.0	2.0	62.8	4.8
6	51.7	40.3	21.0	3.8	1.8	57.6	4.3
7	56.0	46.5	20.0	4.0	2.0	63.9	4.6
8	63.4	47.7	17.0	4.0	2.0	63.1	4.8
9	63.7	47.9	17.0	4.0	2.0	64.8	4.5
10	59.6	45.3	22.0	3.9	1.9	62.8	4.6
11	63.2	48.6	20.0	4.0	2.0	65.5	4.9
12	63.3	50.3	27.0	4.1	2.1	66.3	4.9
13	50.5	45.0	17.0	4.0	2.0	62.4	4.6
14	56.8	44.7	31.0	3.9	1.9	61.3	4.7
15	59.6	45.5	19.0	4.0	2.0	61.4	4.9
16	55.1	39.7	18.0	3.8	1.8	56.7	4.4
17	66.2	48.8	52.0	4.1	2.1	63.6	5.0
18	66.2	48.8	52.0	4.1	2.1	63.6	5.0
19	60.2	43.3	14.0	3.9	1.9	60.8	4.5
20	69.7	47.3	21.0	4.0	2.0	64.7	4.7
21	68.3	50.3	18.0	4.1	2.1	66.8	4.7
22	60.1	46.6	17.0	4.0	2.0	62.6	4.7
23	70.4	51.4	19.0	4.1	2.1	67.0	4.8
24	61.0	44.9	15.0	4.0	2.0	61.1	4.7
25	63.5	47.0	23.0	4.0	2.0	63.9	4.8
26	67.5	45.4	19.0	3.9	1.9	62.7	4.5
27	60.2	41.8	17.0	3.9	1.9	58.3	4.5
28	72.8	51.1	21.0	4.1	2.1	65.7	5.1

Appendix Table A.4. Recurrence Quantitative Analysis measures during the **attacking phase**.

Team	RR	DET	Lmax	L	ENTR	LAM	TT
1	13.5	32.0	13.0	3.6	1.6	54.2	4.3
2	18.1	33.6	13.0	3.7	1.6	56.2	4.2
3	10.8	31.1	11.0	3.7	1.6	53.8	4.2
4	17.4	35.0	15.0	3.8	1.7	56.8	4.6
5	16.4	25.2	30.0	3.5	1.4	49.8	4.2
6	13.1	25.0	21.0	3.6	1.5	49.7	4.1
7	14.9	28.1	10.0	3.6	1.5	52.3	4.0
8	15.6	26.7	9.0	3.5	1.3	49.1	4.0
9	20.0	34.3	16.0	3.7	1.6	55.8	4.2
10	11.7	29.9	17.0	3.6	1.5	52.2	4.3
11	18.4	33.6	15.0	3.7	1.7	55.5	4.4
12	14.8	42.9	21.0	3.9	1.9	63.6	4.7
13	17.2	40.2	14.0	3.9	1.9	60.8	4.5
14	12.0	36.0	31.0	3.8	1.8	55.8	4.5
15	15.7	37.0	13.0	3.8	1.7	55.8	4.5
16	16.0	32.4	16.0	3.7	1.6	52.9	4.3
17	18.2	34.5	19.0	3.7	1.6	53.1	4.3
18	15.0	36.7	24.0	3.9	1.8	56.8	4.8
19	15.0	32.6	11.0	3.7	1.6	55.5	4.2
20	20.2	38.5	21.0	3.8	1.8	60.3	4.5
21	30.6	39.1	16.0	3.8	1.8	59.9	4.5
22	7.8	32.6	11.0	3.8	1.8	56.0	4.4
23	31.6	41.5	13.0	3.9	1.9	60.2	4.6
24	7.5	32.5	10.0	3.7	1.6	53.1	4.3
25	18.0	37.2	23.0	3.8	1.8	58.4	4.6
26	18.4	35.5	13.0	3.7	1.7	57.5	4.3
27	12.0	31.4	11.0	3.7	1.6	51.5	4.3
28	25.1	39.9	21.0	3.9	1.9	58.7	4.8

Appendix Table A.5. Recurrence Quantitative Analysis measures during the **defending phase**.

Team	RR	DET	Lmax	L	ENTR	LAM	TT
1	16.4	28.3	14.0	3.6	1.5	49.0	4.2
2	11.5	26.3	11.0	3.5	1.4	48.9	4.2
3	15.8	33.1	18.0	3.8	1.7	55.6	4.5
4	9.4	28.4	14.0	3.6	1.6	51.5	4.2
5	14.0	27.4	18.0	3.6	1.5	51.4	4.3
6	13.0	19.0	8.0	3.4	1.2	40.1	3.9
7	13.3	27.1	10.0	3.6	1.5	49.3	4.3
8	16.3	32.2	13.0	3.6	1.6	55.0	4.3
9	12.5	31.1	13.0	3.6	1.5	54.2	4.1
10	18.9	31.6	17.0	3.6	1.5	53.9	4.3
11	13.6	34.9	17.0	3.8	1.8	57.2	4.6
12	17.2	33.4	27.0	3.7	1.7	54.2	4.4
13	9.5	27.7	10.0	3.6	1.5	48.8	4.0
14	16.9	33.7	13.0	3.7	1.7	53.9	4.4
15	15.6	36.2	17.0	3.8	1.8	55.9	4.7
16	13.0	29.0	13.0	3.6	1.5	48.5	4.0
17	15.0	36.7	24.0	3.9	1.8	56.8	4.8
18	18.2	34.5	19.0	3.7	1.6	53.1	4.3
19	15.3	29.2	13.0	3.6	1.6	49.3	4.3
20	15.0	29.8	11.0	3.6	1.6	51.1	4.3
21	7.9	28.5	15.0	3.5	1.4	51.0	4.2
22	25.2	35.2	12.0	3.7	1.7	54.9	4.3
23	7.6	34.7	10.0	3.7	1.7	56.6	4.3
24	26.0	33.7	11.0	3.8	1.7	53.4	4.4
25	14.7	27.6	12.0	3.5	1.4	49.7	4.1
26	15.7	28.2	14.0	3.6	1.5	49.9	4.1
27	18.8	30.3	13.0	3.6	1.5	51.0	4.1
28	12.8	34.8	12.0	3.6	1.6	56.9	4.3
