# Supplementary material for: Exploring the recurrent states of football teams’ tactical organization on the pitch during Brazilian official matches
Source: PLoS One. 2024 Aug 12;19(8):e0308320. doi: 10.1371/journal.pone.0308320 (PMC11318918; doi:10.1371/journal.pone.0308320)
Supplement: Attachment — Submitted filename: Editor and Reviewer comments_Plos_One_rev1-rtorres.docx [file pone.0308320.s002.docx]

**Plos One**

**“Exploring the recurrent states of football teams' tactical organization on the pitch** **during Brazilian official matches”**

**Submission ID: PONE-D-23-37093**

May 20^th^, 2024

**Response to Reviewers**

According to the Editor's recommendations, we are resubmitting the paper “Exploring the recurrent states of football teams' tactical organization on the pitch (New title: Exploring the recurrent states of football teams' tactical organization on the pitch during Brazilian official matches”). We would like to thank the editor and the reviewers for their suggestions, which have contributed positively to the quality of the study.

All the suggestions pointed out by the reviewers were accepted and we highlighted all changes on our manuscript in coloured text. We have also included point-by-point responses to the reviewers’ comments.

**Plos One**

**“Exploring the recurrent states of football teams' tactical organization on the pitch** **during Brazilian official matches”**

**Submission ID: PONE-D-23-37093**

|  | **ASSOCIATE EDITOR’S COMMENTS** | **RESPONSE** |
| --- | --- | --- |
| 1 | Thank you for submitting your manuscript to PLOS ONE. After careful consideration, we feel that it has merit but does not fully meet PLOS ONE’s publication criteria as it currently stands. Therefore, we invite you to submit a revised version of the manuscript that addresses the points raised during the review process.  When submitting your revision, we need you to address these additional requirements.  Please ensure that your manuscript meets PLOS ONE's style requirements, including those for file naming. The PLOS ONE style templates can be found at  https://journals.plos.org/plosone/s/file?id=wjVg/PLOSOne_formatting_sample_main_body.pdf and  <https://journals.plos.org/plosone/s/file?id=ba62/PLOSOne_formatting_sample_title_authors_affiliations.pdf>.  Thank you for stating the following financial disclosure:  [This work was supported by the Brazilian National Council for Scientific and Technological Development (CNPq) under grants \#401004/2022-8, \#200290/2022-3, and \#305997/2022-0.].  Please state what role the funders took in the study. If the funders had no role, please state: ""The funders had no role in study design, data collection and analysis, decision to publish, or preparation of the manuscript.""  If this statement is not correct you must amend it as needed.  Please include this amended Role of Funder statement in your cover letter; we will change the online submission form on your behalf.  Thank you for stating the following in the Acknowledgments Section of your manuscript:  [This work was supported by the Brazilian National Council for Scientific and Technological Development (CNPq) under grants \#401004/2022-8, \#200290/2022-3, and \#305997/2022-0.]. We note that you have provided funding information that is not currently declared in your Funding Statement. However, funding information should not appear in the Acknowledgments section or other areas of your manuscript. We will only publish funding information present in the Funding Statement section of the online submission form.  Please remove any funding-related text from the manuscript and let us know how you would like to update your Funding Statement. Currently, your Funding Statement reads as follows:    [This work was supported by the Brazilian National Council for Scientific and Technological Development (CNPq) under grants \#401004/2022-8, \#200290/2022-3, and \#305997/2022-0.].  Please include your amended statements within your cover letter; we will change the online submission form on your behalf.  Please include your full ethics statement in the ‘Methods’ section of your manuscript file. In your statement, please include the full name of the IRB or ethics committee who approved or waived your study, as well as whether or not you obtained informed written or verbal consent. If consent was waived for your study, please include this information in your statement as well. | We would like to thank the editor for the suggestions and for the opportunity to address all the comments and questions.  In the current version of the manuscript, we carefully addressed all raised issues. Point-by-point responses are provided below, and associated modifications are highlighted in the document.   - We have edited the manuscript accordantly with PLOS ONE’s style requirements. - All the requirements concerning the financial disclosure were addressed. - Ethics Committee information were included. |

|  | **Reviewer 1 - COMMENTS** | **RESPONSE** |
| --- | --- | --- |
| 1 | Overall, the article is well written and the topic is interesting. The authors need to make major changes before considering for publication.  Title: Exploring the recurrent states of football teams’ tactical organization on the pitch. Please, modify the title. In my opinion, you are generalising the results of this paper to a broader audience while you just have a sample of 28 Brazilian teams. | We appreciate the reviewer’s suggestions. All the suggestions were taken into consideration.  -----  Thank you for the suggestion about the title. Our intention with the terms “Exploring the recurrent states” was to avoid generalisation and to emphasise the relevance of the recurrence plot as a method to investigate the dynamical properties of tactical-based time series. However, we understand your concern about generalising the results. Therefore, we emphasise that the study was developed with Brazilian matches. Thus, the new title is: ‘Exploring the recurrent states of football teams' tactical organization on the pitch during Brazilian official matches’. |
| 2 | Introduction  L68: please, replace soccer by football. This is the term you have used throughout the manuscript. | Thanks. The term was replaced accordingly. |
| 3 | L12-14: Please, add a citation to this sentence. Also, I suggest taking a look at a systematic review which was recently published and related to your study (https://pubmed.ncbi.nlm.nih.gov/35173369/) for your introduction | Thank you for the suggestion. A reference was inserted for L12-14.  Thank you for the indication of the systematic review. We added this reference in the introduction and discussion sections. |
| 4 | L26-L28: You are introducing the concept of variability here and this term is very important. Can you please add any information explaining what you mean by variability? If you decide to introduce concepts like entropy, please make sure that everyone understands what you are talking about. | Thank you for this suggestion. We have included a definition of variability analysis, highlighting some methods traditionally investigated in sport analytics, including entropy. |
| 5 | L46, L54: Please, remove this abbreviation (RPs). You only mention it twice. | The abbreviation was removed. |
| 6 | L47: Please, remove this abbreviation (RQA). You only mention it once. | The abbreviation was removed. |
| 7 | L62: Explain what you mean by technical performance. This is a very important topic in your study. | We thank the reviewer for the suggestion. In this case, we represented technical performance by traditional game-related statistics as passes performed, shots to goal, goals scored, etc. We included this information when we first mentioned ‘technical performance’. |
| 8 | L67: Please, avoid the recurrent use of “we” when writing a scientific document. Please, correct throughout. | We removed most of the first-person voice in the manuscript, remaining clear sentences in self-reference, following the APA manual which recommends the use of first person in scientific writing. |
| 9 | L67-L73: In my opinion, this information is part of the methods. You may add a section in the methods section in which you talk about the study design or experimental approach to the problem. | This paragraph was reformulated, and the information was concentrated on the methods section, in a subsection for study design. Thank you for this suggestion. |
| 10 | L78: Based on my previous comment, I think that you may leave only the information related to the participants and remove the “data collection” as this could be part of the study design. | Based on your suggestion, we presented general information about study design and removed “data collection” from the subsection head. |
| 11 | L81: data provided by (8)? Please, explain. | Sorry about this confusing sentence. We intend to explain that we used the same data of the study numbered as 8. The sentences of this section were reformulated. Thank you. |
| 12 | L81: How were the data collected? | Additional information was included about DVideo software and data collection. |
| 13 | L86: Please, correct the mistake (the the) | Done. Thank you. |
| 14 | L103: Remove the abbreviation MFD from here and from the equation. | The abbreviation was removed. |
| 15 | L111: defined as the following equation. Please, correct. | The sentence was corrected. |
| 16 | L175, L177: I suggest adding “technical” to …performance indicators… | The term was added to the suggested sentences. |
| 17 | L227: you talk about performance indicators here but this could be understood as tactical as well. I guess it would be “technical performance indicators” | Agreed. The term “technical” was added in the sentence. |
| 18 | L364-L365: Please, expand the practical applications section. This is a poor way to end such an interesting article. You are basically saying what we may already know. Please, think about how your results can have an impact on the field. | Thank you for this suggestion. We have included some clear practical applications, combining with the review that you suggested for the introduction. |
| 19 | References: I found a few mistakes (e.g., avoid mixing uppercase/lowercase in all words, if you choose to add DOI, add all of them when it is possible, etc.) so please, review and follow journal’s guidelines. | All the references were updated. Thank you for the detailed check. |

|  | **Reviewer 2 - COMMENTS** | **RESPONSE** |
| --- | --- | --- |
| 1 | The main focus of the present study was to explore the variability of surface area shapes of football teams on the pitch from recurrence plots and recurrence quantitative analysis, and the association with the performance indicators during the matches,' presents a well-written and innovative investigation. The literature background provides a strong foundation, and the application of recurrence analysis to football matches is a fresh approach. However, to strengthen the paper for publication, a few areas require further clarification. The methods section would benefit from more technical details, and the discussion could be expanded on how these findings translate into actionable insights for coaches and analysts. | We appreciate the reviewer’s suggestions and detailed reading of our manuscript. All the suggestions were taken into consideration, especially the methods and discussion sections.  ----- |
| 2 | Introduction The introduction section is well written. No comments to add. | We appreciate the comments. |
| 3 | Methods 1. L79: Concerning the 14 matches, did you apply any criteria in order to choose them? (red cards for example) | Thank you for concern. The matches were selected by convenience, considering that the datasets were provided for us from previous studies. All the 14 matches are complete and with no red cards or injuries that may have resulted in longer periods with less than 10 outfield players for each team. We have included this information in the methods section. |
| 4 | L80: The 366 players were outfield players? Have you excluded goalkeepers? | No, we did not exclude the goalkeepers from tracking but from the surface area representation only. |
| 5 | L85: Please provide the Ethics committee number | We have included the Ethics committee number |
| 6 | L88-90: Its not clear if the The DVideo software was used manually or automatically identified the actions. | The DVideo software was used to manual register the technical actions of the players during the football matches. We highlighted this information in the manuscript. |
| 7 | L93-44: If there was manual registration (actions, success/no success and responsible player) why you provided the inter- and intra-rater agreement for the system and you didn't conduct new reliability tests? | We agree with the reviewer. Indeed, we evaluated the reliability for the manual register procedure, i.e., the inter and intra-rater agreement. The sentence was reformulated. Thank you very much. |
| 8 | L168-170: You should provide this information in a different paragraph titled "Technical Performance Indicators" or something similar. Its irrelevant to the statistical analysis. Moreover, the first sentence "The technical performance during attacking and defending phases were defined by the nine indicators" needs clarification. You need to provide definitions for the indicators chosen. How you define the "wrong pass", "correct pass", "tackles performed" etc | We have included a new subsection for the technical performance indicators, and we have presented their definitions. |
| 9 | L169: Replace "right pass" with "correct pass" throughout the manuscript. | Done. Thank you. |
| 10 | L177: Why you included the "opponent wrong pass"indicator to defending phase? There could be numerous reasons for the wrong pass, that don't concern the behavior of the defending team. | We partially agree with the reviewer. Although we understand that several reasons may lead to a wrong pass, the same interpretation may be made for all technical action. In other words, even correct actions, such as passes, shots on goal, are dependent on several circumstances that include the teammates and opponent players. However, we associated each indicator to the principles that guide the team action while attacking and defending. Considering that the defending principles are to regain ball possession or to lead the opponent to perform a mistake, we understand that opponent wrong passes were associated with a defending phase, i.e., the defending team has success in its principle. However, we completely understand your concern about the generalisation of this indicator, and we have added this point to the limitations of the study. |
| 11 | Results The results section is well presented. No comments to add. | We appreciate the comments. |
| 12 | Discussion 1.L274-275: The attacking phase includes right passes. I think this sentence needs re-writing. | This sentence was reformulated. Thank you for the detailed review. |
|  | L345-348: Is it possible at the moment to have this fast feedback live during matches at the bench? I believe the last word “performance” should be modified because its an overwhelming statement. You took into account specific performance indicators that maybe are not representative of the performance as a “whole”. Moreover, you should try to enrich your practical implication section, in order to provide coaches and analysts specific “take-home” messages that I am afraid that are not present in this version. | Thank you for this interesting question. Nowadays, with recent advances of player tracking, it would be possible to provide live feedback to coaches during the match. However, that was not our intention. We mentioned fast feedback as a possibility of to quantify the recurrence states of the tactical organization during the first half of the match, for example, to present the coaches and players during interval time. This feedback would explain to staff and players how variable and unpredictable, or how ‘rigid’ their tactical organization was during the match. We reformulated these sentences.  We also addressed your suggestion (and reviewer´s 1 suggestion) about to enrich the practical implications. We presented some examples for training sessions based on the results presented. Thank you. |
|  | L349-356: Based on the comments before concerning Methods section, you should add more limitations. | We have included the limitation about the technical performance indicators and that the outcome may be interpreted with caution. |
|  | L364-365: Again be careful with your statement considering recurrence plot as a tool during football matches. Can you use it live? | Nowadays, with recent advances of player tracking, it would be possible to provide live feedback to coaches during the match. However, we have decided to remove considering that specific championships may restrict the access to such information during the match. Thank you. |
